# Supplementary material for: Proteomic profiling of a Sporobolomyces yeast reveals global responses to UV-B–induced oxidative stress
Source: PLoS One. 2026 Feb 11;21(2):e0333568. doi: 10.1371/journal.pone.0333568 (PMC12893587; doi:10.1371/journal.pone.0333568)
Supplement: Attachment — Submitted filename: Response to Reviewers.docx [file pone.0333568.s004.docx]

Katherine A. Borkovich, Ph.D.
Academic Editor
PLOS ONE

7^th^ January 2026

Re: PONE-D-25-49810

Dear Dr Borkovich

Dear Editor,

My co-authors and I were pleased to receive two highly relevant and constructive reviewer reports on our manuscript. Both reviewers found that the study generated an interesting and valuable proteomic dataset, while also raising closely aligned concerns—principally that the manuscript required substantial shortening, restructuring, and improved focus across all major sections. In particular, the Discussion needed to be rewritten to directly interpret and integrate the results, rather than presenting an extensive literature review.

We have now addressed all reviewer comments and journal requests in full and are pleased to upload a detailed, point-by-point response outlining how each concern has been resolved. Given the extent of the revisions, the changes were implemented in three stages:

1. **Revised Manuscript with Track Changes 1**: This version incorporates all requested typographical and formatting requirements.
2. **Revised Manuscript with Track Changes 2**: This version reflects major structural revisions, including substantial reorganization of the text, a revised title to moderate the rhetoric as requested by both reviewers.
3. **Clean Revised Manuscript**: Here, we updated references, and the inclusion of figure legends and tables within the main text in accordance with journal style. This final version presents the fully revised manuscript with all track changes removed, reflecting the complete set of revisions in a clear and reader-ready format.

We hope that these comprehensive revisions meet the expectations of both the reviewers and the journal. We thank you for the opportunity to revise our manuscript and look forward to your decision.

Yours sincerely,

Paul F. Long PhD

On behalf of the authors

Journal Requirements:

Comment 1: Please ensure that your manuscript meets PLOS ONE's style requirements, including those for file naming.

Response: This has now been done.

Comment 2: Please include your tables as part of your main manuscript and remove the individual files. Please note that supplementary tables (should remain/ be uploaded) as separate "supporting information" files.’

Response: This has now been done.

Comment 3: PLOS requires an ORCID iD for the corresponding author in Editorial Manager on papers submitted after December 6th, 2016.

Response: This has now been done.

Comment 4: Thank you for stating the following financial disclosure: United Kingdom Medical Research Council (MRC Doctoral Training Program Grant G82144A). Please state what role the funders took in the study. If the funders had no role, please state: "The funders had no role in study design, data collection and analysis, decision to publish, or preparation of the manuscript." If this statement is not correct you must amend it as needed. Please include this amended Role of Funder statement in your cover letter; we will change the online submission form on your behalf.

Response: The funding statement has been removed from the Acknowledgements Section, and we kindly ask that you amend the online submission form to include the following statement: ‘This work was supported by the United Kingdom Medical Research Council (MRC Doctoral Training Program Grant G82144A). The funders had no role in study design, data collection and analysis, decision to publish, or preparation of the manuscript.’

Comment 5: Thank you for stating the following in the Acknowledgments Section of your manuscript: We thank Dr Howard G Wildman from Microbial Management Systems Australia for his advice on yeast isolation during the early stages of this work. This work was supported by the United Kingdom Medical Research Council (MRC Doctoral Training Program Grant G82144A). We note that you have provided funding information that is not currently declared in your Funding Statement. However, funding information should not appear in the Acknowledgments section or other areas of your manuscript.

Response: Please see our response to Comment 4.

Comment 6: We note that your Data Availability Statement is currently as follows: All relevant data are within the manuscript and its Supporting Information files. Please confirm at this time whether or not your submission contains all raw data required to replicate the results of your study.

Response: This has now been done and uploaded and S1.

7. We note that you have included the phrase “data not shown” in your manuscript. Unfortunately, this does not meet our data sharing requirements. PLOS does not permit references to inaccessible data.

Response: This phrase has been removed.

8. If the reviewer comments include a recommendation to cite specific previously published works, please review and evaluate these publications to determine whether they are relevant and should be cited. There is no requirement to cite these works unless the editor has indicated otherwise.

Response: No recommendation to cite previously published work has been recommended.

Reviewer #1:

Comment 1: The manuscript by Gaseca et al. presents a proteomic analysis of UV-B–induced stress responses in a UV-tolerant Sporobolomyces strain and generates a dataset that is potentially useful, albeit essentially descriptive in nature. In its current form, however, the work remains largely correlative and falls well short of supporting the very strong causal and evolutionary claims made in the title and abstract, particularly the assertion that an Nrf2/Yap1-like bZIP protein “drives” the UV-induced oxidative stress response and exemplifies an evolutionarily conserved mechanism. The experimental design (limited biological replication, lack of direct ROS or oxidative-damage measurements) and the purely observational nature of the proteomic analysis do not allow the authors to move beyond identifying a candidate regulator and associated protein signatures; they certainly do not justify mechanistic language implying necessity, sufficiency, or functional conservation with the animal Nrf2 pathway. Substantial, data-driven revision is therefore required—either by adding targeted functional validation of the candidate bZIP factor (e.g. genetic perturbation, localisation and downstream target analyses) and strengthening the quantitative proteomic/statistical framework, or by significantly toning down the rhetoric throughout and explicitly reframing the manuscript as a descriptive proteomic survey of UV-B responses.

Response: The title, abstract, and main body of text has been significantly toned down so that the manuscript is a descriptive proteomic survey of the UV-B response.

Comment 2: In addition to these substantive issues, the manuscript also contains several basic and rather elementary formatting, nomenclature and focus problems that further detract from its presentation. For example,

(1) at line 39, the qualifier “sp.” is incorrectly italicised, whereas standard taxonomic convention requires genus and species names to be in italics but rank qualifiers such as sp./spp. to remain in roman type;

Response: Corrected

(2) the layout of several sections (e.g. lines 183–188, 210–220, 299–330, 357–366, 414–424) is highly unconventional for a scientific article, reading more like bullet-point notes than continuous academic prose;

Response: These have been corrected into continuous prose

(3) multiple places in the manuscript (e.g. lines 654–657; 764, 771, 798, 815, 863, 871, 872, 875) still display unresolved Word cross-reference errors such as “Error! Reference source not found.”, indicating that the citation and cross-referencing system has not been properly checked prior to submission;

Response: We apologise for this oversight. The errors were not present in the original Word document and arose during the conversion to a single PDF file. We will carefully review the file conversion process when submitting the revised manuscript. We sincerely thank the reviewer for bringing this issue to our attention.

(4) the quality, numbering and labelling of the final figures and tables are far below publication standard and severely compromise readability—for instance, only “Figure 1” and “Figure 11” appear, “Figure 1” is reused several times without clear distinction, and one figure is even labelled “Figure Error! No text of specified style in document.”;

Response: We have revised the labelling of figures and text in accordance with Comment 1 of the Journal Requirements. We are confident that the updated labelling now meets the journal’s standards, and we thank the reviewer for their helpful comment.

(5) with respect to species names, Rhodosporidium toruloides has been reclassified as Rhodotorula toruloides and the genus Rhodosporidium is no longer in current use, so the entire manuscript should be carefully checked and updated to reflect the current, accepted taxonomy;

Response: Corrected

(6) an inordinate amount of space is devoted to animal systems and to Saccharomyces cerevisiae—especially the latter—far beyond what would be reasonable as background, whereas the stated subject of the manuscript is UV stress responses in a Sporobolomyces yeast, so the balance of the Introduction and Discussion should be substantially adjusted so that the narrative is clearly centred on UV-B–induced responses and on the Sporobolomyces system actually investigated here;

Response: We thank the Reviewer for their valuable suggestions and we have now substantially reduced the length of the manuscript (including the number of references from 150 to 57) and have adjusted the narrative to become clearly centred on the UVB induced responses actually investigated.

(7) the use of italics for biological and technical terms is often incorrect or inconsistent—for instance, Latin-derived expressions such as ex vivo (and similarly in vivo, in vitro, in situ, etc.) should consistently appear in italics, while gene/protein symbols and other specialist terminology should follow standard formatting conventions. These issues collectively suggest that the manuscript requires much more careful technical editing and proofreading before it can be considered for publication.

Response: We thank the reviewer for their valuable comments. The manuscript has been carefully revised to ensure consistent use of italics throughout, and we appreciate the reviewer’s attention to this detail.

Reviewer #2:

Comment 1: The Introduction is overly detailed. This makes it difficult for the reader to identify the key context needed to understand the study. The authors should significantly shorten the Introduction and ensure that only relevant background information is retained.

Response: We have shortened the Introduction to focus on the most relevant background information, as suggested by the reviewer. This revision ensures that the key context needed to understand the study is clear and concise, in line with the approach recommended in Reviewer 1, Comment 1.

Comment 2: Each subsection of the Results should begin with a short introductory sentence explaining the purpose of the analysis. All figures in the manuscript are labelled as “Figure 1,” which needs correction. In Figure 1, the abbreviation “BY” is used but never defined. The description of MudPIT results is unnecessarily fragmented, with proteins divided into multiple categories, each discussed in separate paragraphs. This part should be condensed substantially to highlight the main trends without excessive repetition.

Response: We thank the reviewer for these detailed suggestions. The Results section has been thoroughly revised to incorporate all recommendations. Each subsection now begins with a concise introductory sentence outlining the purpose of the analysis. All figures have been correctly labelled, and the abbreviation “BY” in Figure 1 has been defined. The description of the MudPIT results has been consolidated, with protein data presented in a more integrated format to highlight the main trends while minimizing repetition. This has considerably improved the flow of this section as well as reducing the length of the text including the number of references from 150 to 57.

Comment 3: The Discussion requires a major rewrite. Concerns include excessive length: Over 30 pages is not acceptable for this type of dataset; it should be condensed to a maximum of 3 pages. Lack of focus: much of the text provides general literature review rather than discussing the authors’ specific findings.

Response: The Discussion has been extensively revised to focus on the main findings of our study, minimising general literature review. These changes have considerably reduced the length of the Discussion to approximately three A4 pages, single-spaced, in accordance with the reviewer’s recommendation.

Comment 4: Methodology included in Discussion (L662–668): Methods should not appear in this section.

Response: References to methodology in the Discussion (L662–668) have been removed as part of the revision, in accordance with Comment 3.

Comment 5: Terminology correction: At L676, the term “ROS” should replace “RS.”

Response: Corrected throughout the text.

Comment 6: Overly long, difficult-to-read sentences: For example, the sentence across lines 674–678 should be rewritten for clarity. Chaotic section on stress responses: The discussion of toxic metal responses lacks structure and does not integrate well with the main argument. This section should be reorganized.
Yap transcription factor section (L739–758): The discussion of Yap factors in S. cerevisiae is too extensive, especially given that no homologs were found in Sporobolomyces. This information should be condensed or removed.

Response: We have thoroughly revised the Discussion section in response to the reviewer’s previous comments. We sincerely thank the reviewer for their suggestions, which have greatly improved the clarity and readability of the manuscript.

Comment 7: Scope of conclusions: In lines 699–704, conclusions are presented as if they apply only to yeast, although similar UV-B-induced antioxidant responses occur in human cells. The authors should consider whether the described mechanisms are more general.

Response: We have revised the Conclusions to emphasize the shared features between yeast and human cells and to present a global, qualitative perspective rather than a mechanistic interpretation, in accordance with Reviewer 1 (Comments 1 and 6) and Reviewer 2 (Comments 3 and 6).
